# Synthesis of acrylic resins for high-solids traffic marking paint by solution polymerization

**DOI:** 10.1080/15685551.2019.1699349

**Published:** 2019-12-11

**Authors:** Maryam Taheri, Mehdi Jahanfar, Kenji Ogino

**Affiliations:** a Graduate School Of Bio-Applications and System Engineering, Tokyo University of Agriculture and Technology, Koganei, Japan; b Assistant Professor of Shahid Beheshti University, Department of Life sciences and, Biotechnology, Tehran, Iran; c Tokyo University of Agriculture and Technology, Material Systems and Engineering, Koganei, Japan

**Keywords:** High﻿-solid traffic marking paint, acrylic resin, organic peroxide initiator, peroxyester, peroxyketal, dialkylperoxide, azo initiator

## Abstract

Fast-drying traffic marking paint comprising a solvent-borne resin, a filler, a pigment and a solvent that is especially suitable for colder ambient (temperatures near freezing) applications, where waterborne traffic paint cannot be used. Acrylic resins based on methyl methacrylate, butyl acrylate, acrylic acid, and styrene were synthesized in different solvents using organic peroxide initiators such as peroxyester, peroxyketal, dialkylperoxide and azo. After polymerization, the molecular weight (M_w_), polydispersity index = PDI (M_w_/M_n_), viscosity, total residual monomer and APHA color were evaluated and results of organic peroxide initiators (t-butyl and t-amyl derivatives) were compared with the azo initiator. The M_w_, PDI, viscosity, mass conversation and APHA color of resins with t-amyl derivatives of organic peroxide initiators are very proper.

## 1. Introduction

In cold and humid weather, traffic marking paint based on aqueous acrylic resin slowly dried which makes more difficult on busy roads and even leads to unfortunate accidents. In this situation, on the roads with high traffic, the interaction between traffic marking paint and glass beads is minimal and limited adhesions, generally within a few months, night reflectivity decrease. Solving this problem, especially for roads with a high volume of traffic is very important. The solvent-based traffic marking paint can be used that quickly dried without remaining the inside portion of the coated paint layer in the undried state. In these situations, high-solid traffic marking paint based acrylic resin with proper viscosity is very applicable.

The development of high-solid paint (HSP) in some countries was necessary to get this goal and meet strict environmental regulations geared to reduce emissions of volatile organic compounds (VOCs) [1–19]. While Europe has lagged the U.S., similar regulations are being considered [18]. Also, in 1998, the Council Directive on the limitation of emissions of VOCs sets out a target for solvent emission reduction in the European Union [19]. This target is an emission cut of at least 50% compared to 1990 emission levels for all industrial activities using organic solvents, namely, car refinishes.

Since white line was first painted on a road in 1911 in Michigan, U.S.A., horizontal road markings became ubiquitous, essential safety feature of modern roads. It was observed that in the city of Kraków, Poland almost all road marking is done with the thin-layer application of a solvent-borne toluene-containing paint. Toluene, an aromatic solvent, is generally recognised as harmful to people and environment; on the other hand, its relatively low price, convenient solubility parameters, and evaporation rate make it a preferred choice for solvent-borne road marking paints, unless banned by a legislation [20].

The topic of troposphere ozone formation caused by road marking paints has been introduced only very recently, with the first brief note done by Scorgie [21] and recently by Burghardt [22,23]. Knowing of extreme air pollution in Kraków, we postulated that an improvement could be achieved by limiting the VOCs emitted from road marking paints and consequently the formed tropospheric ozone. The currently used road marking system and readily available alternative paints are analysed herein. Knowing of extreme air pollution in Kraków, we postulated that an improvement could be achieved by limiting the VOCs emitted from road marking paints and consequently the formed tropospheric ozone. The currently used road marking system and readily available alternative paints are analysed [20].

So, efforts in recent years have been directed toward the production of high-solid traffic marking paints with fast drying time and low emissions of volatile organic compounds, especially in the winter season. In solvent-borne formulations with acceptable solution viscosity, the solid content can be increased by reducing the solvent content; however, the product quality and performance must be maintained in the higher solid content formulations. In order to achieve HSP formulations based on acrylic resin and ensure the lowest viscosity, acrylic polymers with both low molecular weight and a narrow molecular weight distribution (MWD) must be used [13]. However, the required quality standards cannot be compromised, particularly with regard to appearance, physical properties, and performance.

The most common method used to produce low molecular weight acrylic polymers is to employ proper kind and high concentrations (up to 6%) of the initiator, alone or in combination with chain transfer agents, and careful control over the process variables. The initiator plays a dominant role in the free-radical synthesis of these resins. Both the chemical nature of the initiator and the polymerization conditions greatly influence the efficiency of the polymerization and the final resin properties. So, selection of proper initiator results in high-solid resin with low molecular weight, narrow polydispersity, low viscosity, and reduced volatiles. Reported are guidelines to improve acrylic resin properties through a better understanding of initiators.

In classical free-radical chemistry, the initiator is simply treated as ‘R^•^’ and little consideration is given to the chemical nature of the R group, initiator decomposition mechanisms, and the type of radicals produced.

Decomposition rates of initiators are commonly reported in terms of half-life (t _1/2_) time or temperature, i.e., the time at which 50% of the initiator has decomposed at a specified temperature or the temperature at which 50% of the initiator has decomposed at a specified time, respectively. To aid in the selection of the optimum organic initiator and half-life characteristics best suited for a particular polymerization temperature.

To a large extent, the half-life range at which an initiator decomposes determines the application and control overall process efficiency and product quality. In their product bulletins, initiator producers often provide half-life data over a wide range of temperatures. For optimum efficiency, however, an initiator is usually chosen so that the half-life under the reaction conditions is in the range of 10–20 min. This ensures the steady generation of radicals at such a rate that the heat of reaction can be safely contained and a high conversion of monomer to polymer results. It is important to note, however, that initiator half-life data are usually determined in select inert solvents and low initiator concentration. Decomposition rates can be affected by solvent polarity, radical-induced decomposition, and initiator concentration [24]. The temperature activity is not the sole consideration in selecting an initiator for a particular application. Other factors to be taken into account include cost, solubility, safety, efficiency and type of radicals produced, the necessity for refrigerated storage and shipment, compatibility with production equipment, the effect on the final product, and the ability to be activated.

In recent years, organic peroxide initiators have become a dominant factor in the synthesis of low molecular weight, narrow molecular weight distribution acrylic resin [25]. Advances in organic peroxide technology such as t-amyl peroxides have led to a better understanding of the structure/reactivity relationships of the initiator and the effect on resin properties [26,27].

The goal of this study was to determine the optimum polymerization conditions and produce higher solids with low viscosity, and higher quality with a study on the variety kinds of initiators. While these efforts have been partially successful, limitations still exist. Many drawbacks are influenced by differences in free-radical reactivity and associated structural features of the initiator. In this study, azo, peroxyester, peroxyketal and dialkyl peroxide initiators in combination with high concentrations of a chain transfer agent are generally employed. The azo initiator was evaluated against each t-amyl and t-butyl derivatives of organic peroxide initiators at an equivalent active [N].

## 2. Experimental

### 2.1. Materials of acrylic resin

Methyl methacrylate (99%), butyl acrylate (99%), styrene (99%) and 2-mercaptoethanol (98%) were obtained from the Aldrich Chemical Co.

Tert-butyl peroxy-2-ethylhexanoate (Trigonox 21S), Tert-amyl peroxy-2-ethylhexanoate (Trigonox 121), 1,1-di-(tert-butyl peroxy) cyclohexane (Trigonox 22-E50), 1,1-di-(tert-amyl peroxy) cyclohexane (Trigonox 122-C80), Tert-butyl peroxy-3,5,5-trimethylhexanoate (Trigonox 42S), D-t-Butyl Peroxide (Trigonox B) and 2,2ʹ azodi (methyl butyronitrile) (Perkadox AMBN) were obtained from Akzo Nobel Co., and D-t-Amyl Peroxide (Luperox DTA) and Tert-amyl peroxy-3,5,5-trimethylhexanoate (Luperox 570) were obtained from Arkema Co. All the organic peroxides were evaluated at their 0.1-h half-life temperatures and an equal active [O] = 0.42 phm unless otherwise stated (see Table 1) [27,28]. Also, azo initiator was evaluated at an equal active [N] = 0.42 phm. All the t-amyl and t-butyl derivatives of organic peroxide initiators that used in this paper are liquids with excellent solubility. They cover a wide activity range with greater efficiency and yield resins with lower color.

**Table 1.** General and kinetic data of initiators [27,28].

		Half-life Temperature(t_1/2_), °C				
Trade name	Chemical structure	10 HR	1 HR	0.1 HR	Active [O] or [N] %^1^	Weight % (phm)^2^
**Peroxyester**						
Trigonox 21S	Tert-butyl peroxy-2- ethylhexanoate	72	91	113	7.40 [O]	5.67
Trigonox 121	Tert-amyl peroxy-2-ethylhexanoate	73	91	111	6.95 [O]	6.03
Trigonox 42S	Tert-butyl peroxy-3,5,5- trimethylhexanoate	94	114	135	6.95 [O]	6.04
Luperox 570	Tert-amyl peroxy-3,5,5- trimethylhexanoate	100	120	130	6.5 [O]	6.67
**Peroxyketal**						
Trigonox 22-E50	1,1-di-(tert-butyl peroxy) cyclohexane	94	113	134	12.3 [O]	3.41
Trigonox 122-C80	1,1-di-(tert-amyl peroxy) cyclohexane	87	106	126	11.11 [O]	3.78
**Dialkyl Peroxide**						
Trigonox B	D-t-Butyl Peroxide	121	141	164	10.95 [O]	3.83
Luperox DTA	D-t-Amyl Peroxide	123	143	150	9.16 [O]	4.57
**Azo initiator**						
Perkadox AMBN	2,2ʹ azodi (2-methyl butyronitrile)	66	84	104	7.29 [N]	5.76

^a^ At 100% assay

^b^ phm = parts per hundred parts monomer at an active [O] or [N] = 0.42 phm

As shown as in Table 1, that most of the t-amyl and t-butyl derivatives of organic peroxide initiators also offer lower use levels (phm) at an equivalent activity as compared to the azo initiator which may improve the cost efficiency of the process.

Xylene (>99.5%, boiling point = 135°C), toluene (>99.5%, boiling point = 110°C) was obtained from Merck. Solvesso 100 (boiling point = 165–180°C) was obtained from Mehta Petro Refineries Limited.

### 2.2. Materials of traffic marking paint

To prepare traffic marking paint, a copolymer acrylic resin, titanium dioxide pigment, filler, solvent, and additives were used.

Thermoplastic acrylic resin, a binary copolymer of methyl metha acrylate (MMA), butyl acrylate (BA), styrene (ST) and acrylic acid (AA) in the approximate weight ratio of 36:40:23:1 in toluene with different initiators and temperature of reaction (were produced in this study with sample name a-l).

Titanium dioxide pigment is Ti-Pure™ R-900, manufactured by DuPont in America.

The filler is calcium carbonate, manufactured by Lorestan chemical and mineral co. in Iran. The solvent is toluene (>99.5%, Merck). The anti-settling additive is Bentone 34 (Elementis specialties, Malaysia). The dispersing agent is Troysperse CD1 (Troy CO., Canada).

Drop on glass beads is produced by Baztabrah Co. (Shiraz, Iran) medium grading according to BS EN 1423.

### 2.3. Synthesis of acrylic resins

Low viscosity acrylic resins were prepared by conventional free-radical solution polymerization techniques in different initiators. The polymerizations were conducted under nitrogen in a jacketed, stainless steel reactor equipped with a mechanical stirrer, a thermometer with a temperature controller, and reflux condenser. Table 2 presents weight percent of the main components for the preparation of the samples according to the experimental design of the mixture method. The monomer feed and initiator were metered and charged into the reactor containing solvent at a prescribed temperature over a 5-h period. The chain transfer agent coexists in the monomer, prior to or at the initiation of polymerization, in an amount of 1 wt % at the most [30]. Free radicals from the initiator may abstract hydrogens from the acrylic polymer which can lead to chain branching, a broader MWD, and higher solution viscosity. Therefore, chain transfer agents (e.g., Mercaptans) are needed to lower the molecular weight; however, mercaptan chain transfer agents can produce objectionable odors, color, and light instability in the coatings [31].

**Table 2.** Acrylic resin formulations [30].

Raw materials	*Weight percent*
Methyl methacrylate	20
Butyl acrylate	22.5
Styrene	13
Acrylic acid	0.5
2-mercaptoethanol	0.5
Initiator (see Table 1)	3.5
Solvent	40

**Figure 1.**
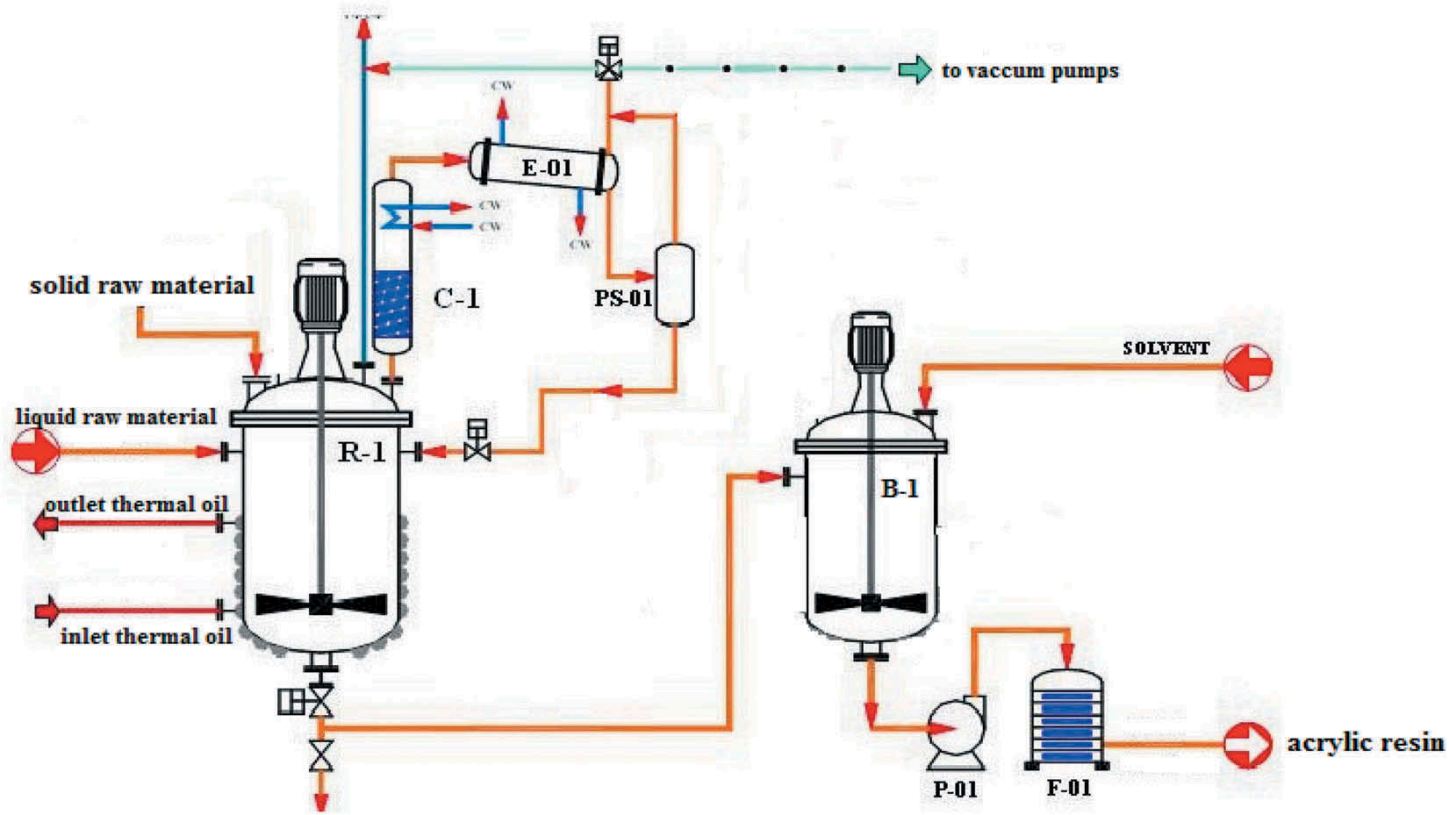
Schematic illustration of the proposed mechanism for acrylic resin synthesis [30].

After the monomer and initiator addition was complete, the polymerization was continued for an additional hour. The monomer to solvent ratio was 3.7 to 1 (80% solids theoretical). Figure 1 illustrates the schematic of the proposed mechanism for the synthesis of acrylic resin. After polymerization was complete, polymer charged into the blender to adjust 60% solids with solvent [30].

### 2.4. Preparation of cold-applied traffic marking paint

**Figure 2.**
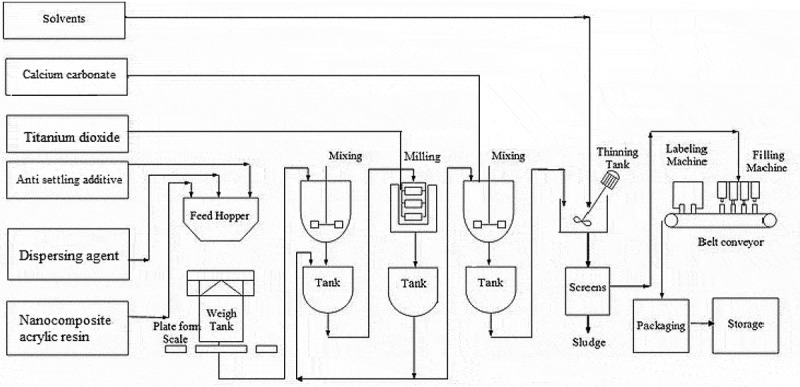
Schematic illustration of the proposed process flow diagram for synthesis [30].

Table 3 presents weight percent of the main components for the preparation of the samples according to the experimental design of the mixture method. Figure 2 illustrates schematically of the proposed process flow diagram for the synthesis of traffic marking paint. Anti-settling additive and the dispersing agent were dispersed in acrylic resin for 10 min. The pigment is then added to the dispersion that is then ball-milled using laboratory KREIS BASKET MILL/KREIS-DISSOLVER (Niemann, Germany).

**Table 3.** A´- L´ cold-applied traffic marking paint formulations [30].

Raw materials	Weight percent
Acrylic resin (sample name a-l)	30
Anti settling additives	0.5
Dispersing Agent	0.2
Titanium dioxide	10
Calcium carbonate	50
Solvent	10

This operation continues until the fineness of grind paint is max 10 microns according to ISO 1524. Then, add gradually calcium carbonate and mix for 15 min, with solvent adjusts the viscosity to 95–100 KU according to ASTM D562 [30].

### 2.5. Characterization of acrylic resins

Tg of polymers were determined with DSC analysis using Mettler ToledoStar System equipment DSC3. To eliminate solvent influence, two runs were scanned, from −20 to 150 ºC, held at this temperature for 10–15 min and run again from – 20 to 150 ºC at a heating rate of 10 K/min under nitrogen flow. The results of the second cycle were used to determine Tg.

Molecular weight and MWD of polymers can be measured by many different methods such as vapor phase osmometry, ultracentrifugation and light scattering, the method used in this paper is gel permeation chromatography (GPC). The advantages of using GPC are: (1) moderate cost, (2) fast analysis time, (3) excellent reproducibility of results, (4) can be applied to a wide variety of solvents and polymers, (5) can be applied to a wide range of molecular weights and (6) good agreement of results, particularly MWD, with results obtained from other techniques. The THF SEC system consists of a Waters 1515 isocratic HPLC pump, a Waters 717 plus auto-sampler, Waters 600E system controller (run by Breeze Version 3.30 SPA) and a Waters in-line degasser AF. A Waters 2414 differential refractometer is connected in series with a Waters 2487 dual-wavelength absorbance UV/Vis detector operating at variable wavelengths. Tetrahydrofuran (THF, HPLC grade, stabilized with 0.125% BHT) is used as mobile phase, at a flow rate of 1ml/min under the operating temperature of 30°C and an injection volume of 100 µl. Two PLgel (Polymer Laboratories) 5 µm Mixed-C (300 x 7.5 mm) columns and a pre-column (PLgel 5 µm Guard, 50 × 7.5 mm) is used [30]. Calibration is done using narrow polystyrene standards ranging from 580 to 2_*_10^6^ g/mol (Agilent Technologies) and all molecular weights were reported as polystyrene equivalents.

The viscosity of acrylic resins was determined using Brookfield DV-II+ Viscometer at 25°C with a 4 spindle rotating at 20 RPM according to ISO 2555 with 50% solid content [30].

**Figure 3.**
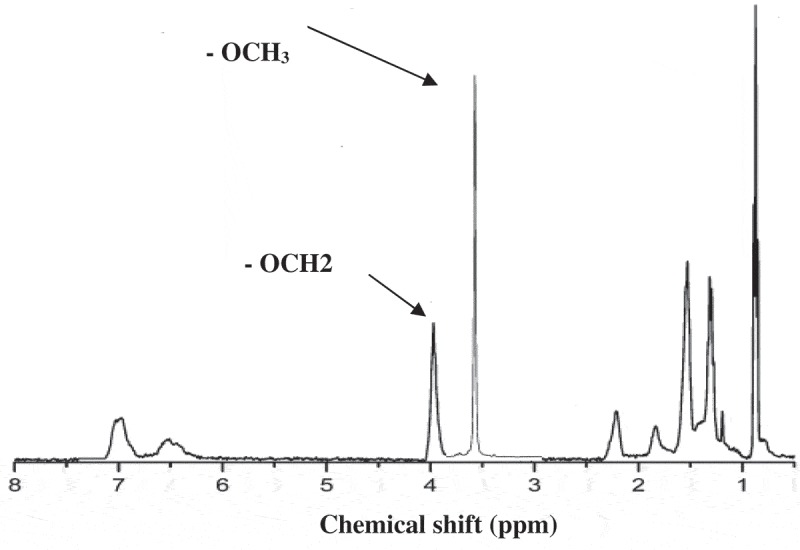
HNMR of acrylic resins.

Mass conversion based on the total polymer in the reaction mixture was measured using gravimetry. The resulting isolated copolymers were analyzed for cumulative polymer composition using a Bruker AMX500 and a Bruker MX300 Fourier Transform NMR spectrometer. The dried polymer was dissolved in deuterated chloroform (≈2% w/v) at room temperature [30]. All spectra exhibited good peak separations for diagnostic signals. Figure 3 illustrates the signal at≈4.0 ppm was due to the – OCH2 group in BA, another signal at≈3.6 ppm was associated with the – OCH3 group of MMA and another signal at≈6.4–7.1 ppm was associated with the aromatic region of ST [30].

The relative mole fractions of monomer bound in the polymer (F_BA_ or F_MMA_) were determined from the areas under the corresponding peaks [30].

The color was determined by APHA color values based on a color test (ASTM-D2849) scale of 5 to 500, in increments of 5.

### 2.6. Characterization of cold-applied traffic marking paint

Non-volatile matter determines the fraction of a paint that is non-volatile at the temperature of the test while volatile solvents are driven off. It is sometimes an approximate measure of the film-forming matter in a paint. Nonvolatile matter determined by Test Method B of ASTM D 1644.

No-pick-up time covers a laboratory test for estimating (after application of traffic marking paint) the drying period required for no-pick-up by the tire of an automobile that determined by Test Method ASTM D 711. No-pick-up time was determined using Model DT-711106 of Gardco Company. The apparatus consists of a cylinder with two 3/8” cross-section diameter ‘O’ rings made of a synthetic rubber meeting the requirements of HK 715 Specification D2000. The rings will retain their flexibility indefinitely. The total weight of the tester including ‘O’ rings is 11 pounds 14 ounces (± 1 oz) (5386 g ± 28 g), which is equivalent to tire pressure of 100 pounds per square inch on the pavement. The rolling ramp furnished is made of aluminum with a 1:6 slope measuring 6” long, 3-1/2” wide and 1” high at the upper slotted end.

Hiding power of traffic paints determines with Procedure A, Method 4121 of U.S. Federal Test Method 141B. Paint when applied at the rate of 10 mils wet film thickness over a Merest Black and White Hiding Power Chart, Form 03-B, shall show complete hiding or give a contrast ratio of not less than 0.98 between the reflectance of the black and of the white chart surfaces as determined by a Hunter Multi-Purpose Reflectometer.

Resistance to wear determined by Test Method ASTM D 968. In the referenced method abrasive is poured on to a dry film on a glass panel until the paint is removed. A typical value for traffic marking paint is 65 L of sand for the removal of a 3-mil (75 µm) dry film.

Water resistance determined by Test Method ASTM D 1647. Apply a 5-mil (130 µm) wet film to a clean glass panel, allow to air dry for 72 h, immerse in reagent water for 24 h, and allow a recovery period of 2 h before examining.

## 3. Results and discussion

### 3.1. Results and discussion of acrylic resins

#### 3.1.1. Mole fraction of monomers and tg study

Table 4 illustrates mole fraction of monomers in acrylic resins (after 20 h of the reaction) and glass transition temperature (Tg) at the reaction temperature 103, 123, 112 and 157 ^°^C that produced with organic peroxide initiators such as peroxyester, peroxyketal, dialkylperoxide and azo. F _MMA_ is the mole fraction of methyl methacrylate unit in acrylic resins; F _BA_ is the mole fraction of butyl acrylate unit in acrylic resins; F _st_ is the mole fraction of styrene unit in acrylic resins. In higher F _MMA_ and F _ST_, Tg of acrylic resins will increase, because the Tg of MMA (100 °C) and ST (105ºC) are higher than the Tg of BA (−54 ºC).

**Table 4.** Mean values of F _MMA_, F **_BA_**, F **_ST_** and Tg of acrylic resins.

Trade name of initiator	Code of resin	Reaction temperature (°C)	F _MMA_	F _BA_	F _ST_	Tg (°C)
**Peroxyesters vs. Azo**						
Trigonox 21S	a	103	39.43	36.06	24.51	17.43
Trigonox 121	b	103	39.57	35.82	24.61	17.83
Perkadox AMBN	c	103	39.12	36.54	24.33	17.10
Trigonox 42S	d	123	39.63	35.72	24.65	17.77
Luperox 570	e	123	39.75	35.53	24.72	18.21
Perkadox AMBN	f	123	36.32	41.09	22.59	13.11
**Peroxyketals vs. Azo**						
Trigonox 22-E50	g	112	39.71	35.59	24.70	17.92
Trigonox 122-C80	h	112	39.79	35.46	24.75	18.25
Perkadox AMBN	i	112	38.05	38.28	23.67	16.00
**Dialkylperoxides vs. Azo**						
Trigonox B	j	157	39.70	35.60	24.70	17.82
Trigonox 201	k	157	39.79	35.45	24.76	18.14
Perkadox AMBN	l	157	35.06	43.12	21.82	10.97

So, main reason is that during the solution polymerization, when the final conversion is low, the monomer with a strong conjugacy is easier to polymerize than the others, styrene is such a hard monomer and the glass transition temperature of its homopolymer is 105 °C, so the Tg of copolymer will be a little higher than usual; on the contrary, when the final conversion is high, the system is able to polymerize according to the expectable proportion.

However, the conversion of polymerization can not reach 100%. In addition, Tg will increase because of the hydrogen bonds formed between the Components [32].

#### 3.1.2 M_w_ study

The primary factors that influence the molecular weight properties of acrylic resins and the efficiency of the polymerization are the initiator concentration, initiator type and radicals produced, the rate of decomposition of the initiator, the polymerization temperature, the solvent type, monomer mix, and feed rate of monomers and initiator. In this study, the initiator concentration and feed rate of monomers and initiator are fixed.

Therefore, the initiator or initiator type in most cases is the dominant factor in controlling and lowering the molecular weight. To a large extent, the decomposition rate at which an organic initiator produces free radicals determines the application temperature and controls the overall polymerization efficiency. In most polymerizations of acrylic monomers, the polymerization temperature is greater than 100 °C and the organic initiator is chosen so that the half-life time under the reaction conditions is in the range of 2 to 20 min.

**Figure 4.**
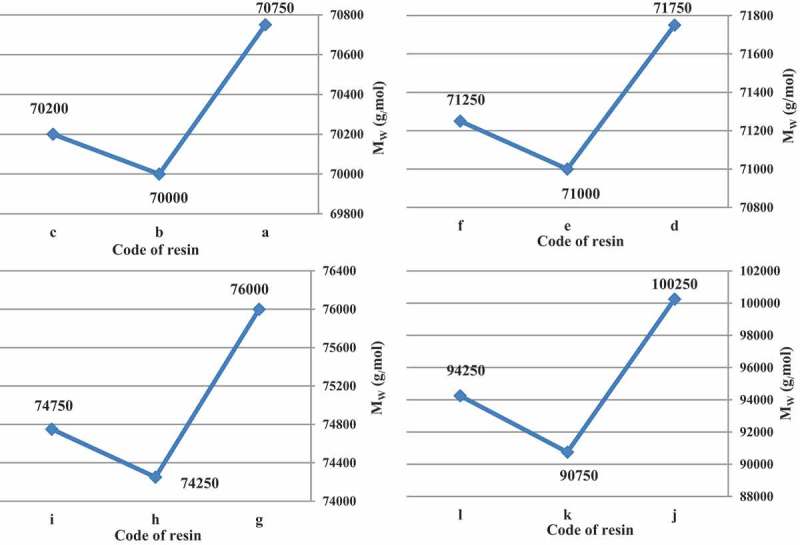
M_W_ of acrylic resins with using Trigonox 21S (a), Trigonox 121 (b), Perkadox AMBN (c), Trigonox 42S (d), Luperox 570 (e), Perkadox AMBN (f), Trigonox 22-E50 (g), Trigonox 122-C80 (h), Perkadox AMBN (i), Trigonox B (j), Trigonox 201 (k), Perkadox AMBN (l).

In all cases, t-amyl derivatives of organic peroxide initiators as compared to their t-butyl derivatives of organic peroxide initiators analogs and also azo initiator produced resins with lower M_w_. Figure 4 illustrates M_w_ of acrylic resins at the reaction temperature 103, 123, 112 and 157 °C that produced with organic peroxide initiators such as peroxyester, peroxyketal, dialkylperoxide and azo.

#### 3.1.3. PDI study

The H-abstracting ability of the free radicals generated from the initiator plays a major role in determining the PDI. Azo initiators decompose to form alkyl radicals, which are more selective and poor in hydrogen (H) abstracting ability [33]. Although organic peroxides initially cleave at the oxygen–oxygen bond, other bond cleavages can and do occur, either simultaneously with or sequencers to the oxygen–oxygen bond dissociation. The relative stability of the R radical determines whether a peroxide undergoes single- or multiple-bond homolysis. In some cases, the PDI was less than 1.5 that utilizing the t-amyl peroxide vs. the t-butyl peroxide and azo initiator produced resins with lower PDI.

**Figure 5.**
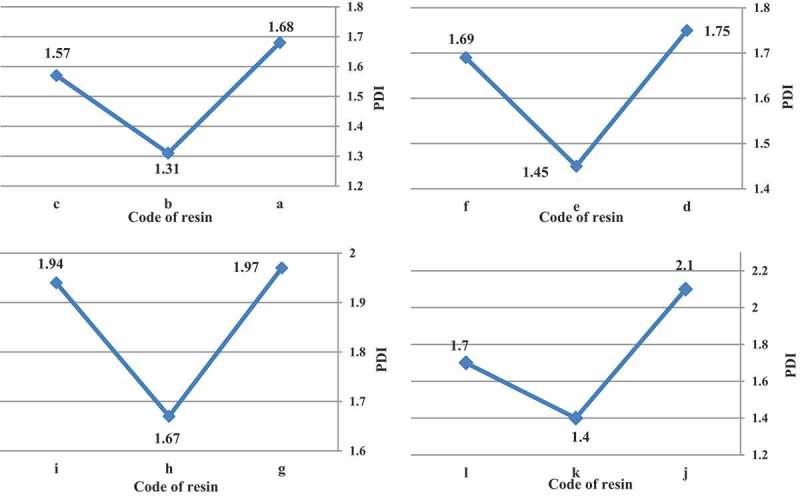
PDI of acrylic resins with using Trigonox 21S (a), Trigonox 121 (b), Perkadox AMBN (c), Trigonox 42S (d), Luperox 570 (e), Perkadox AMBN (f), Trigonox 22-E50 (g), Trigonox 122-C80 (h), Perkadox AMBN (i), Trigonox B (j), Trigonox 201 (k), Perkadox AMBN (l).

Figure 5 illustrates PDI of acrylic resins at the reaction temperature 103, 123, 112 and 157 ^°^C that produced with organic peroxide initiators such as peroxyester, peroxyketal, dialkylperoxide and azo.

#### 3.1.4. Viscosity study

**Figure 6.**
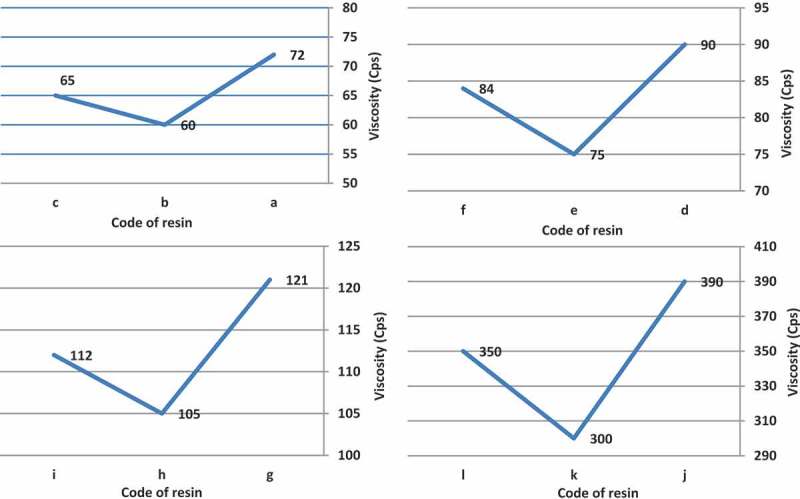
Viscosity of acrylic resins with using Trigonox 21S (a), Trigonox 121 (b), Perkadox AMBN (c), using Trigonox 42S (d), Luperox 570 (e), Perkadox AMBN (f), Trigonox 22-E50 (g), Trigonox 122-C80 (h), Perkadox AMBN (i), Trigonox B (j), Trigonox 201 (k), Perkadox AMBN (l).

In all cases, t-amyl derivatives of organic peroxide initiators as compared to their t-butyl derivatives of organic peroxide initiators analogs and also azo initiator produced resins with lower viscosity. Figure 6 illustrates viscosity of acrylic resins at the reaction temperature 103, 123, 112 and 157 ^°^C that produced with organic peroxide initiators such as peroxyester, peroxyketal, dialkylperoxide and azo.

However, the viscosity of acrylic resins is proportional to the molecular weight of resins according to the Mark-Houwink equation [34,35].

$$\mu =\;K{\left(M.W.\right)}^{a}$$

µ: viscosity of acrylic resins

K and a: constants derived

From Figure 6, it is clear that the viscosity and average molecular weight of the acrylic resins increased with increase in the amount of BA content in the compositions.

#### 3.1.5. Mass conversation study

Most polymerizations are conducted at temperatures greater than 100ºC and the half-life of the azo is lower rather than other initiators, so the rate of azo ‘s decomposition is very fast and thus the efficiency of the polymerization is reduced. The higher the bond dissociation energy, the less stable (more reactive) is the corresponding radical that is formed by removing the hydrogen atom. Thus, the phenyl radical is significantly more reactive than the alkyl radicals. The relative stability of alkyl radicals is in the order of tert-alkyl >sec-alkyl >n-alkyl > methyl. The methyl radical is about as reactive as an alkoxy radical.

In all cases, t-amyl derivatives of organic peroxide initiators as compared to their t-butyl derivatives of organic peroxide initiators analogs and also azo initiator produced resins with higher mass conversation.

**Figure 7.**
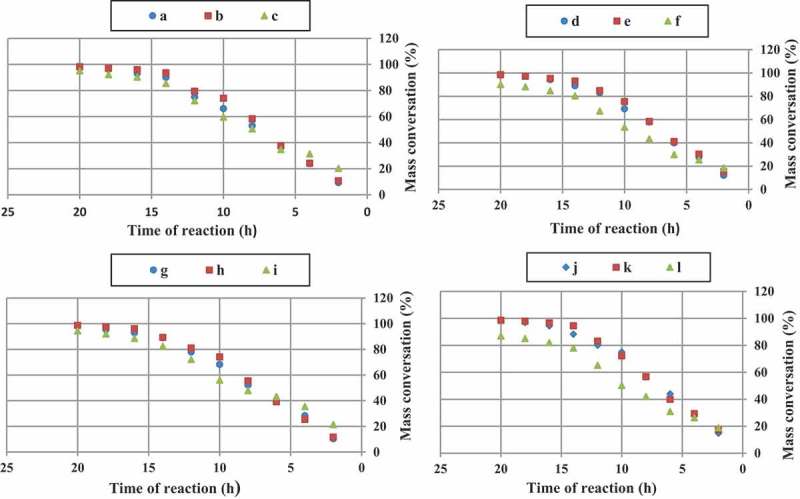
Mass conversation of acrylic resins with using Trigonox 21S (a), Trigonox 121 (b), Perkadox AMBN (c), Trigonox 42S (d), Luperox 570 (e), Perkadox AMBN (f), Trigonox 22-E50 (g), Trigonox 122-C80 (h), Perkadox AMBN (i), Trigonox B (j), Trigonox 201 (k), Perkadox AMBN (l).

Figure 7 illustrates mass conversation of acrylic resins at the reaction temperature 103, 123, 112 and 157^°^C that produced with organic peroxide initiators such as peroxyester, peroxyketal, dialkylperoxide and azo.

#### 3.1.6. Color study

The APHA color is directly related to the structural differences of the initiators tested, the differences in the rate of decomposition or half-life and illustrate the importance in considering the free-radical type as an integral part of the experimental design. The APHA color values for all initiators were less than 35 compared to the azo-produced resins which were greater than 200. The nitrile functionality of the azonitrile initiator can yield byproduct compounds that generate color in acrylic resins.

**Figure 8.**
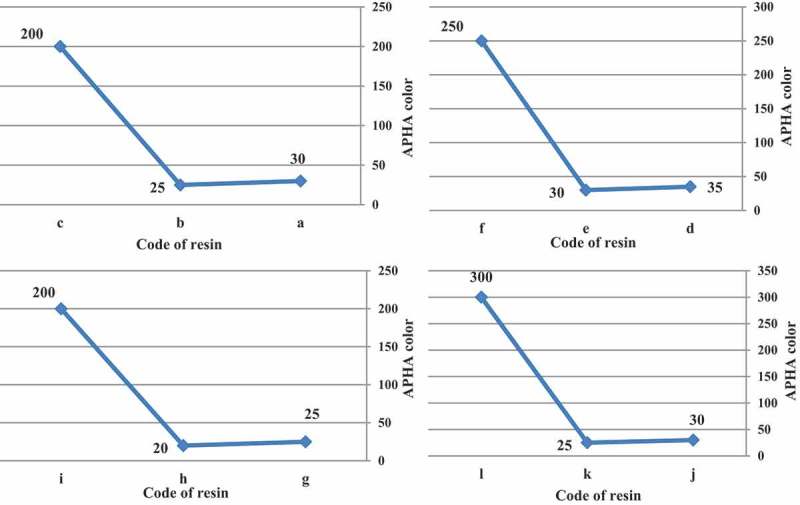
APHA color of acrylic resins with using Trigonox 21S (a), Trigonox 121 (b), Perkadox AMBN (c), Trigonox 42S (d), Luperox 570 (e), Perkadox AMBN (f), Trigonox 22-E50 (g), Trigonox 122-C80 (h), Perkadox AMBN (i), Trigonox B (j), Trigonox 201 (k), Perkadox AMBN (l).

In all cases, t-amyl derivatives of organic peroxide initiators as compared to their t-butyl derivatives of organic peroxide initiators analogs and also azo initiator produced resins with lower color. Figure 8 illustrates APHA color of acrylic resins at the reaction temperature 103, 123, 112 and 157 ^°^C that produced with organic peroxide initiators such as peroxyester, peroxyketal, dialkylperoxide and azo.

Totally, in all cases, t-amyl derivatives of organic peroxide initiators produced resins had lower M_w_, PDI, viscosity and APHA color and higher mass conversation as compared to their t-butyl derivatives of organic peroxide initiators analogs and also azo initiator.

### 3.2. Results and discussion of cold-applied traffic marking paint

#### 3.2.1. Non-volatile matter study

**Figure 9.**
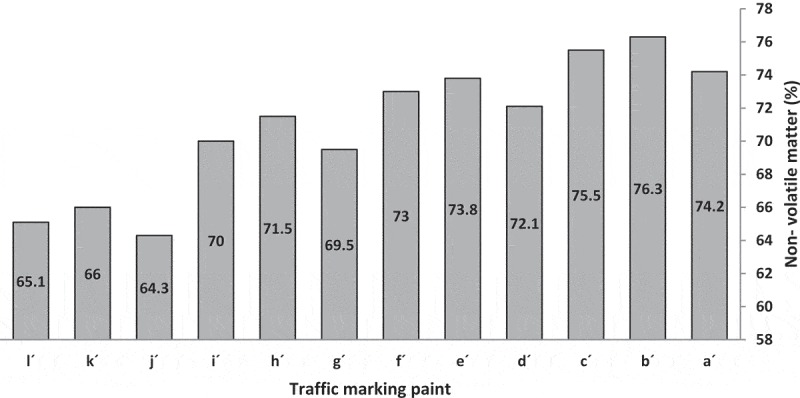
Non-volatile matter of traffic marking paints.

The percent nonvolatile matter indicates the amount of material remaining after the solvent evaporates and is a measure of the film solids. Figure 9 illustrates the nonvolatile matter of traffic marking paints with code a´-l´. Resins with lower viscosity produced paint with higher nonvolatile matter because to adjust the same viscosity they need less solvent (volatile matter).

#### 3.2.2. No-pick-up time study

The drying time of a traffic marking paint is particularly important because it determines how quickly a lane can be opened to the free flow of traffic without the paint being transferred to adjacent pavement. Solvent evaporating occur in two below mechanisms:

- In the first part, solvent mass decreases very quickly. During this fast regime, the solvent mass fraction at the air/film interface remains high (>0.25), as does the diffusion coefficient; thus a significant diffusion and evaporation of solvent take place [36].
- In the second part, solvent concentration, although low at the air/film interface, remains high (<0.25), diffusion becomes too slow to allow sufficient regeneration of the solvent near the interface. The diffusion coefficient changes according to the polymer/solvent ratio. The evaporation flux of solvent becomes very weak and the concentration gradient decreases [36].

**Figure 10.**
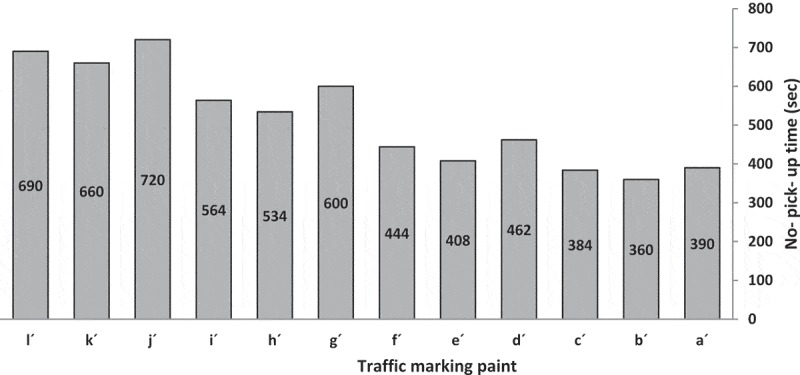
No-pick-up time of traffic marking paints.

Figure 10 illustrates the no-pick-up time of traffic marking paints with code a´-l´. Traffic marking paints with higher nonvolatile matter has shorter no-pick-up time, because in this situation there is less solvent to get out of the paints.

#### 3.2.3. Hiding power study

Hiding power or opacity is a measure of the ability of a paint to hide the substrate. It varies, naturally, with the thickness and Non-volatile matter of the applied film. Table 5 illustrates the hiding power of traffic marking paints with code a´-l´. Traffic marking paints with higher nonvolatile matter has higher hiding power, because at this situation, in same viscosity, the amount of titanium dioxide is higher.

**Table 5.** Hiding power of traffic marking paints.

		Results of hiding power (contrast ratio)							
		Measurement number						Mean	Standard devision
Code of traffic marking paint	a´	98	98.2	98.1	98	98.2	98.1	98.1	0.2
	b´	99.1	99.2	99.2	99.2	99.2	99.3	99.2	0.2
	c´	98.7	98.6	98.5	98.5	98.7	98.6	98.6	0.2
	d´	96	96.1	96	95.9	96	96	96	0.2
	e´	97.3	97	97.4	97.4	97	97.1	97.2	0.4
	f´	96.4	96.6	96.5	96.4	96.6	96.5	96.5	0.2
	g´	93.3	93.3	93.1	92.9	93	93	93.1	0.4
	h´	95	94.7	95.1	95.1	94.7	94.8	94.9	0.4
	i´	93.5	93.5	93.5	93.4	93.6	93.5	93.5	0.2
	j´	87.4	87.3	87.4	87.8	87.6	87.5	87.5	0.5
	k´	89.1	89.3	89.2	89.1	89.3	89.2	89.2	0.2
	l´	88.1	88.1	88.2	88	88.2	88	88.1	0.2

#### 3.2.4. Resistance to wear study

Resistance to wear is a measure of the ability of the dried film to withstand wear from traffic and from objects rolled or pulled across the surface that determined by Test Method ASTM D 968. Table 6 illustrates resistance to wear of traffic marking paints with code a´-l´. Traffic marking paints with higher nonvolatile matter has better resistance to wear, because, at the same wet film thickness, their dry film thickness becomes thicker.

**Table 6.** Resistance to wear of traffic marking paints.

		Results of resistance to wear (L)							
		Measurement number						Mean	Standard devision
Code of traffic marking paint	a´	71.9	71.9	72.3	72.2	72.1	72.2	72.1	0.4
	b´	75.1	74.9	75	75	75	75	75	0.2
	c´	74	73.9	74	73.9	73.8	73.8	73.9	0.2
	d´	69	68.9	69	69	69.1	69	69	0.2
	e´	71.5	71.3	71.7	71.6	71.4	71.5	71.5	0.4
	f´	70.1	70.1	70.3	70.1	70.3	70.3	70.2	0.2
	g´	65.2	65.2	65.1	64.9	65	65.1	65.1	0.4
	h´	67.8	67.9	67.9	67.9	68.1	67.8	67.9	0.3
	i´	66	66	65.9	66	66.1	66	66	0.2
	j´	52	52	52	51.9	52	52.1	52	0.2
	k´	54	53.8	54	53.9	54	54.1	54	0.3
	l´	53.4	53.2	53.4	53.2	53.4	53.2	53.3	0.2

#### 3.2.5. Water-resistance study

This property is important to traffic marking paint because they are frequently exposed to rain or condensation on bridges that determined by Test Method ASTM D 1647. Table 7 illustrates water resistance of traffic marking paints with code a´-l´. Traffic marking paints with higher nonvolatile matter has better water resistance, because, at the same wet film thickness, the amount of acrylic resin is higher, dry film thickness becomes thicker. This will eventually lead to better water resistance.

**Table 7.** Water resistance of traffic marking paints.

Code of traffic marking paint	Results of the water test
a´	Not visible affected
b´	Not visible affected
c´	Not visible affected
d´	Whitening disappears within 20 min
e´	Not visible affected
f´	Not visible affected
g´	Whitening does not disappears within 20 min, but disappears within 2 h
h´	Whitening disappears within 20 min
i´	Whitening disappears within 20 min
j´	Whitening does not disappears within 2 h, but disappears within 24 h
k´	Whitening does not disappears within 2 h, but disappears within 24 h
l´	Whitening does not disappears within 2 h, but disappears within 24 h

## 4. Conclusion

Initiators can have a variety of characteristics depending on their chemical structure and reactivity. The reactivity of the initiators depends on the initiator group configuration and on the type of substituents. T-butyl derivatives of organic peroxide are the most stable radicals in termination and propagation stage of a polymeric chain. In all cases, the results showed that t-amyl derivatives of organic peroxide initiators as compared to their t-butyl derivatives of organic peroxide initiators and also azo initiator produced resins with low M_w_, low PDI, high conversation, low APHA color and lower solution viscosities. Resins with lower viscosity produced traffic marking paints with high nonvolatile matter that leads to traffic paints with shorter no-pick-up time, higher hiding power and better resistance to wear and water.
